# Loss of zebrafish *pkd1l1* causes biliary defects that have implications for biliary atresia splenic malformation

**DOI:** 10.1242/dmm.049326

**Published:** 2023-10-11

**Authors:** Rouknuddin Q. Ali, Anne Meyer-Miner, Marie David-Rachel, Fiona J. H. Lee, Benjamin J. Wilkins, Saul J. Karpen, Brian Ciruna, Anand Ghanekar, Binita M. Kamath

**Affiliations:** ^1^ Program in Developmental & Stem Cell Biology, The Hospital for Sick Children, Toronto, ON M5G 0A4, Canada; ^2^ Department of Molecular Genetics, The University of Toronto, Toronto, ON M5S 1A8, Canada; ^3^ Department of Pathology and Laboratory Medicine, The Children's Hospital of Philadelphia, Philadelphia, PA 19104, USA; ^4^ Department of Pediatrics Emory, University School of Medicine and Children's Healthcare of Atlanta, Atlanta, GA 30322, USA; ^5^ Division of General Surgery, University Health Network, Toronto, ON M5C 2C4, Canada; ^6^ Department of Surgery, The Hospital for Sick Children, Toronto, ON M5G 1X8, Canada; ^7^ Department of Surgery, University of Toronto, Toronto, ON M5T 1P5, Canada; ^8^ Division of Gastroenterology, Hepatology and Nutrition, The Hospital for Sick Children, Toronto, ON M5G 1X8, Canada; ^9^ Department of Pediatrics, University of Toronto, Toronto, ON M5G 1X8, Canada

**Keywords:** PED6, CRISPR/Cas9, Laterality, Ciliopathy, Bile duct

## Abstract

Biliary atresia is a fibroinflammatory neonatal disease with no effective therapies. A subset of cases (10-20%) is associated with laterality defects – labeled biliary atresia splenic malformation (BASM) syndrome. Recently, whole-exome sequencing of patients with BASM identified deleterious variants in *PKD1L1*. PKD1L1 is involved in left-right axis determination; however, its role in cholangiocytes is unknown. We generated the *pkd1l1^hsc117^* allele using CRISPR/Cas9 mutagenesis in zebrafish to determine the role of Pkd1l1 in biliary development and function. Wild-type and mutant larvae were assessed for laterality defects, biliary function and biliary tree architecture at 5 days post fertilization. *pkd1l1^hsc117^* mutant larvae exhibited early left-right patterning defects. The gallbladder was positioned on the left in 47% of mutants compared to 4% of wild-type larvae. Accumulation of PED6 in the gallbladder, an indicator of hepatobiliary function, was significantly reduced in *pkd1l1^hsc117^* mutants (46%) compared to wild-type larvae (4%). *pkd1l1^hsc117^* larvae exhibited fewer biliary epithelial cells and reduced density of the intrahepatic biliary network compared to those in wild-type larvae. These data highlight the essential role of *pkd1l1* in normal development and function of the zebrafish biliary system, supporting a role for this gene as a cause of BASM.

## Teaser

**Summary:** *PKD1L1*-linked biliary atresia is a serious childhood liver disease with no effective therapies. Deletion of zebrafish *pkd1l1* results in impaired function and abnormal development of bile ducts in the liver.

## INTRODUCTION

Biliary atresia (BA) is a progressive, idiopathic, fibroinflammatory disease affecting extra- and intra-hepatic bile ducts, leading to cholestasis, portal fibrosis and, ultimately, biliary cirrhosis (Mack et al., 2012). BA is the leading indication for liver transplantation in children. The underlying etiopathogenesis of BA is unknown, despite decades of study, and, hence, there is a lack of mechanistic understanding or effective therapies. Current hypotheses for potential etiologies include genetic predisposition, a disorder of bile duct development, an environmental factor such as a virus or toxin, immune dysregulation, or a combination of one or more of these contributors. BA occurs as an isolated hepatobiliary finding in the majority of patients, whereas non-hepatic developmental malformations are seen in approximately 20% of cases (Schwarz et al., 2013). This group with associated anomalies is labeled as BA splenic malformation (BASM) as these patients exhibit a spectrum of laterality and patterning defects that include a variety of manifestations of heterotaxy, structural heart disease, intestinal malrotation or polysplenia.

Previous studies support a potential role for aberrant cilia or ciliary signaling in the etiopathogenesis of heterotaxy syndromes (Li et al., 2019), including BASM. The presence of laterality defects in patients with BASM strongly suggests a contribution from ciliary signaling, as it is well established that laterality specification and patterning during embryogenesis are principally determined by signals interpreted by cilia (Field et al., 2011; Kamura et al., 2011). In the liver, cholangiocytes (but not hepatocytes) possess primary cilia. The primary cilium on the apical surface of each cholangiocyte incorporates environmental signaling and ion flux, thus contributing to bile formation and responses within cholangiocytes (LaRusso and Masyuk, 2011). Intrahepatic cholangiocyte cilia are less abundant, significantly shorter and abnormally oriented in patients with BASM (Sheflin-Findling et al., 2012). Furthermore, analysis of extrahepatic bile ducts taken from infants with BA show an absence of cholangiocyte cilia (Karjoo et al., 2013).

The ciliary gene polycystic kidney disease 1 like 1 (*PKD1L1*) was recently found to be biallelically mutated in a portion of patients with BASM via a whole-exome sequencing approach, providing a new opportunity to explore genetic contributions to BASM (Berauer et al., 2019). Although this study demonstrated PKD1L1 expression in the bile duct epithelium in human liver tissue, the role of PKD1L1 in biliary tract function and development remains to be determined. PKD1L1 is expressed on primary cilia and co-localizes and interacts with polycystic kidney disease 2 (PKD2) and polycystic kidney disease 2-like 1 (PKD2L1) proteins. There are limited known functional roles for PKD1L1, except that it forms heterodimers with PKD2L1 (PKD1L1:PKD2L1), which form an intraciliary calcium channel (DeCaen et al., 2013; Delling et al., 2013). PKD1L1:PKD2 interactions are essential for proper signaling in the embryonic node and serve as a basis for early left-right (L-R) axis determination during development (Field et al., 2011; Kamura et al., 2011; Delling et al., 2013). Mutations in *PKD1L1* are associated with laterality defects in mouse models and heterotaxy in humans; however, other than the association with BASM, biliary tract anomalies have not been described in any model (Vogel et al., 2010; Field et al., 2011; Grimes et al., 2016; Vetrini et al., 2016; England et al., 2017). The goal of this study was to investigate the role of Pkd1l1 in zebrafish, a well-described model of biliary tract development (Cofer and Matthews, 2014).

## RESULTS

### Generation of *pkd1l1^hsc117^* zebrafish using CRISPR/Cas9

**Fig. 1.**
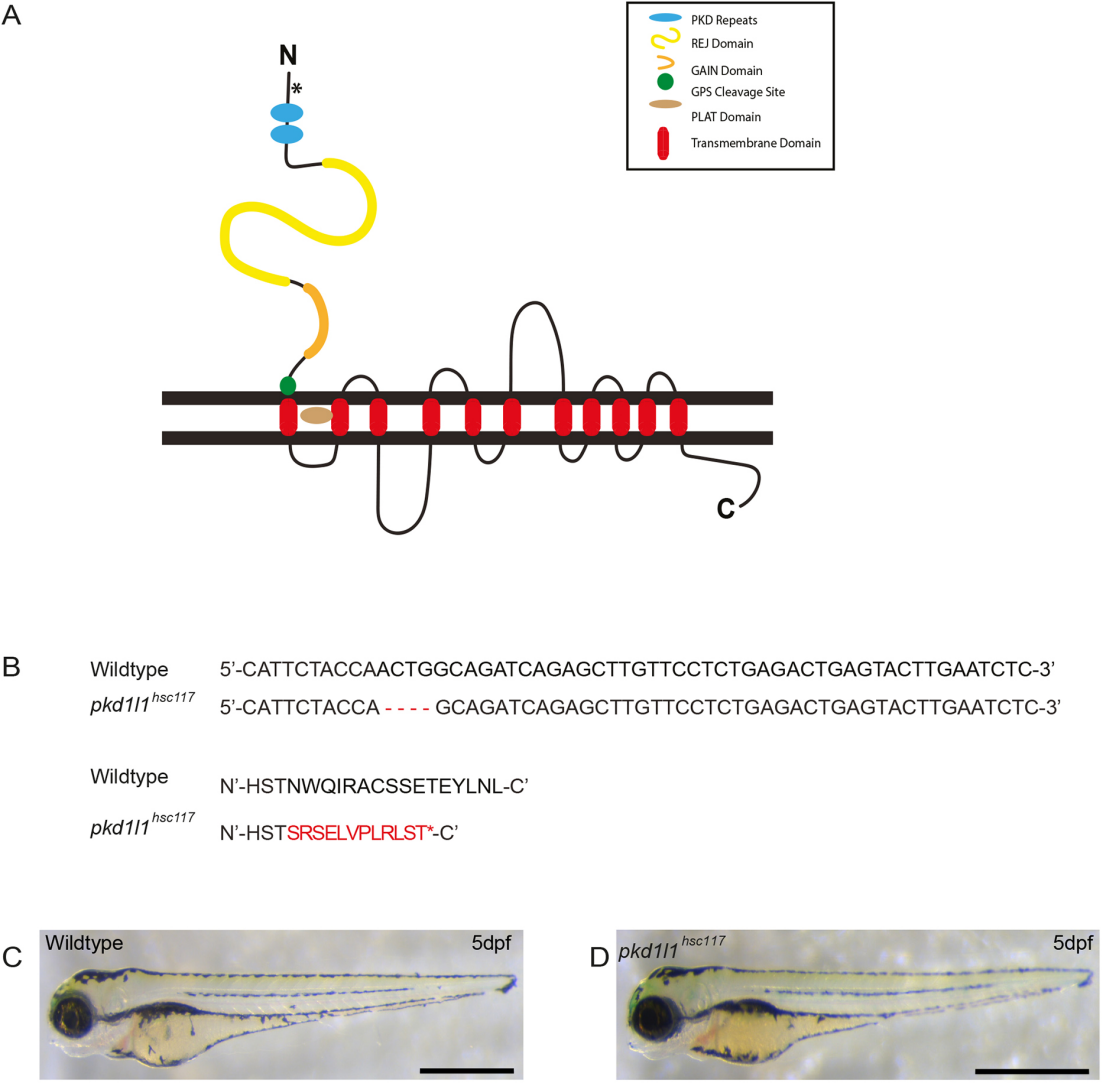
**Generation of *pkd1l1^hsc117^* zebrafish mutants.** (A) Pkd1l1 is a large membrane-spanning protein with multiple transmembrane domains, with a predicted length of 2153 amino acids (England et al., 2017). The asterix indicates the CRISPR/Cas9 deletion target site. (B) Pairing of nucleotide and amino acid sequences of wild-type and CRISPR/Cas9-derived mutants, revealing mutant F0 zebrafish with a 4 bp deletion, resulting in a premature stop codon. (C,D) F1 embryos were raised, heterozygotes identified and F1 heterozygotes were incrossed to generate *pkd1l1^hsc117^* mutant embryos. At 5 dpf, wild-type (C) and *pkd1l1^hsc117^* (D) zebrafish are morphologically indistinguishable. Images are representative of 50 fish (*n*=50). Scale bars: 1 mm.

Injection of *Cas9* mRNA and single guide (sg)RNAs targeting deletion of 4 bp in the third exon of *pkd1l1* resulted in a frameshift and incorporation of premature termination codons predicted to lead to early protein truncation and a strong hypomorphic or loss-of-function allele (Fig. 1A,B). Zygotic *pkd1l1^hsc117^* mutant fish were viable and fertile, with no gross morphological defects (Fig. 1C,D). Incrosses of homozygous *pkd1l1^hsc117^* mutant animals were therefore used to generate maternal and zygotic mutant embryos for further phenotypic analysis.

### *pkd1l1^hsc117^* mutant zebrafish demonstrate laterality defects

**Fig. 2.**
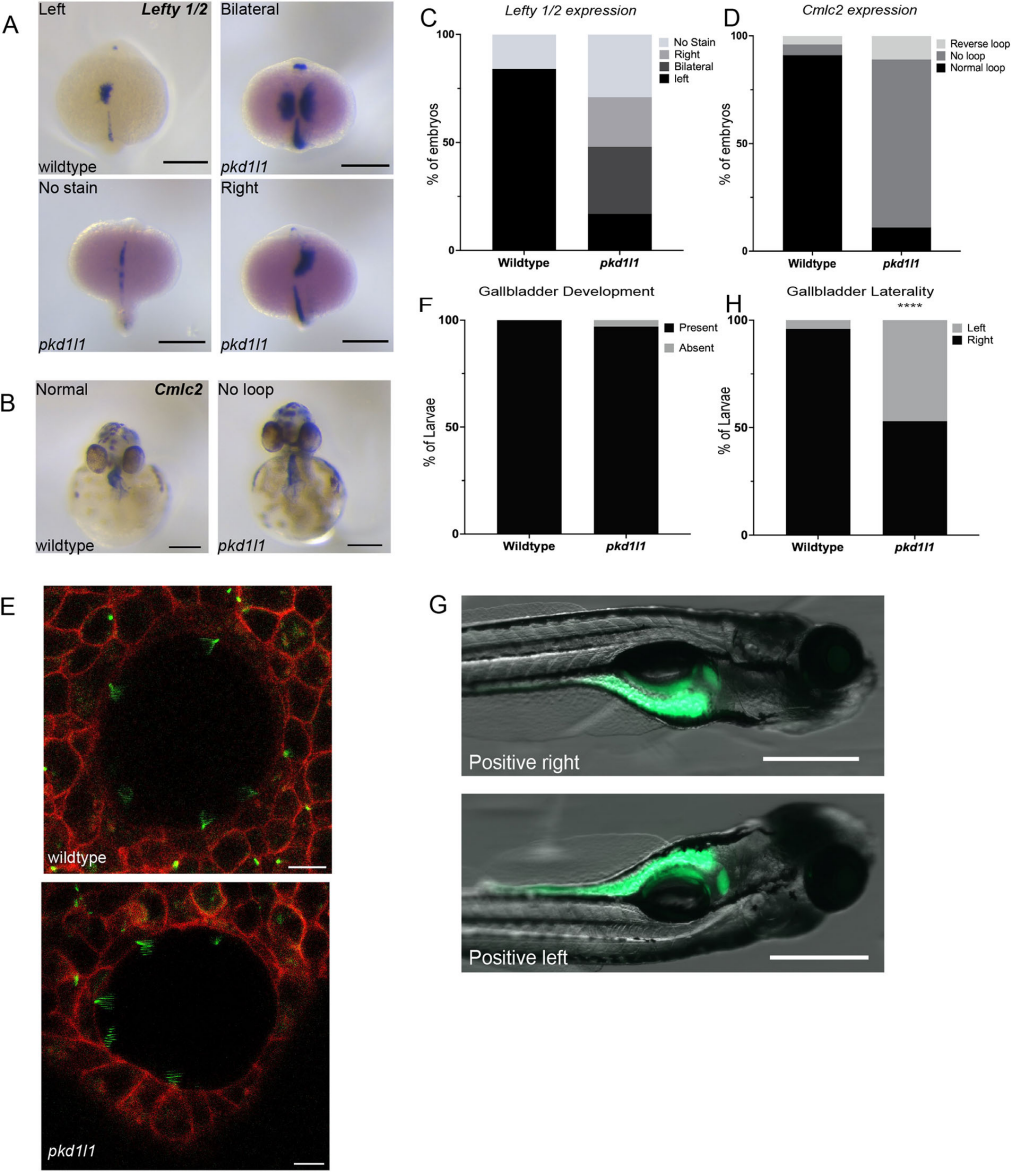
***pkd1l1* mutant embryos demonstrate laterality defects.** (A,B) Whole-mount RNA *in-situ* hybridization demonstrating *lefty 1* and *lefty 2* (*lefty 1/*2) expression in wild-type and *pkd1l1* mutant embryos (dorsal view at 19 hpf) (A) and *cmlc2* expression in wild-type and *pkd1l1* mutant embryos (ventral view at 48 hpf) (B). Scale bars: 0.25 mm. (C) *lefty 1/2* expression was only demonstrated on the left side in wild-type embryos (*n*=45), with expression being dispersed among the left, right and bilateral sides in *pkd1l1* mutant embryos (*n*=48). (D) *cmlc2* expression demonstrated normal looping in the majority of wild-type embryos (*n*=74), but in *pkd1l1* mutant embryos (*n*=36), looping was primarily absent, and a few *pkd1l1* mutant embryos showed either normal or reverse looping. (E) Confocal images of the KV of 10-16 somite wild-type (*n*=5) and *pkd1l1* mutant (*n*=7) embryos demonstrate the presence of motile cilia. Arl-GFP is shown in green and membrane-localized RFP (memRFP) in red. Scale bars: 10 μm. (F) Quantification of the presence of the gallbladder showed no significant differences in gallbladder development in wild-type (*n*=33) and *pkd1l1* mutant (*n*=39) larvae. (G) Position of the gallbladder as seen from right and left lateral views. Scale bars: 500 µm. (H) *pkd1l1* mutations cause laterality defects in gallbladder positioning as demonstrated by an increased frequency of left-sided gallbladders in *pkd1l1* mutant (*n*=156) compared to wild-type (*n*=166) zebrafish. ****P<0.0001 (Fisher's exact test).

Pkd1l1 was previously found to be critical for L-R patterning and asymmetric organ positioning in mouse and medaka (Field et al., 2011; Kamura et al., 2011). In zebrafish, England et al. (2017) detailed the expression of *pkd1l1* during early development, specifically in the laterality-defining organ Kupffer's vesicle (KV). As such, we first screened *pkd1l1^hsc117^* embryos for laterality defects (Fig. 2A-D). At 19 h post fertilization (hpf), expression of *lefty 1* (*lft1*) and *lefty 2* (*lft2*) (markers of L-R patterning) was present on the left lateral plate mesoderm (LPM) in 84% of wild-type embryos. In contrast, only 18% of *pkd1l1^hsc117^* embryos expressed *lefty 1/2* on the left LPM, 22% expressed *lefty 1/2* on the right LPM and 30% demonstrated bilateral *lefty 1/2* expression (Fig. 2A,C). Next, we examined looping of the heart using the marker *cardiac myosin light chain 2* (*cmlc2*, also known as *myl7*). At 48 hpf, the cardiac tube looped normally in 92% of wild-type embryos, with only 4% exhibiting reversed or no looping. In contrast, only 10% of *pkd1l1^hsc117^* mutant exhibited normal cardiac looping, whereas 80% of mutant embryos developed with medial placement of the atrium and ventricle (no looping) and 10% of *pkd1l1^hsc117^* mutants displayed reversed looping (Fig. 2B,D). Taken together, these data confirm the presence of laterality defects in *pkd1l1^hsc117^* mutant zebrafish. This L-R patterning defect prompted us to examine the formation and behavior of motile cilia in *pkd1l1^hsc117^* mutant KVs. Live imaging of Arl13b-GFP (a cilia axoneme reporter) and membrane-localized red fluorescent protein (memRFP) localization at somite stages 10-14 revealed the presence of functional motile cilia in both wild-type and *pkd1l1^−/−^* mutant KVs, as demonstrated by the blurred, fanlike appearance of motile cilia produced by line-averaged image acquisition (Fig. 2E) (Borovina et al., 2010). In concordance with previous observations in medaka (Kamura et al., 2011) and mice (Field et al., 2011) that highlight a lack of phenotypic differences in the KV/nodal cilia of wild-type versus *pkd1l1* mutant embryos, our results suggest an action of Pkd1l1 downstream of nodal flow.

### *pkd1l1^hsc117^* mutant zebrafish display defects in gallbladder positioning

To examine the role of Pkd1l1 in the zebrafish hepatobiliary system, we quantified the presence of a gallbladder in 5 days-post-fertilization (dpf) wild-type and *pkd1l1^hsc117^* mutant larvae by whole-mount immunostaining with the 2F11 antibody, a monoclonal antibody that labels secretory cells in the zebrafish intestinal epithelium and bile ducts. There was no significant difference in the presence of a gallbladder in wild-type (100%) or *pkd1l1^hsc117^* mutant (97%) larvae (Fig. 2F); however, we observed left-sided gallbladder positioning in a greater percentage of *pkd1l1^hsc117^* mutant larvae (47%) compared to wild-type larvae (4%) (Fig. 2G,H). These data suggest that Pkd1l1 is involved in gallbladder positioning but does not affect gallbladder development.

### *pkd1l1^hsc117^* mutant zebrafish have impaired biliary function

**Fig. 3.**
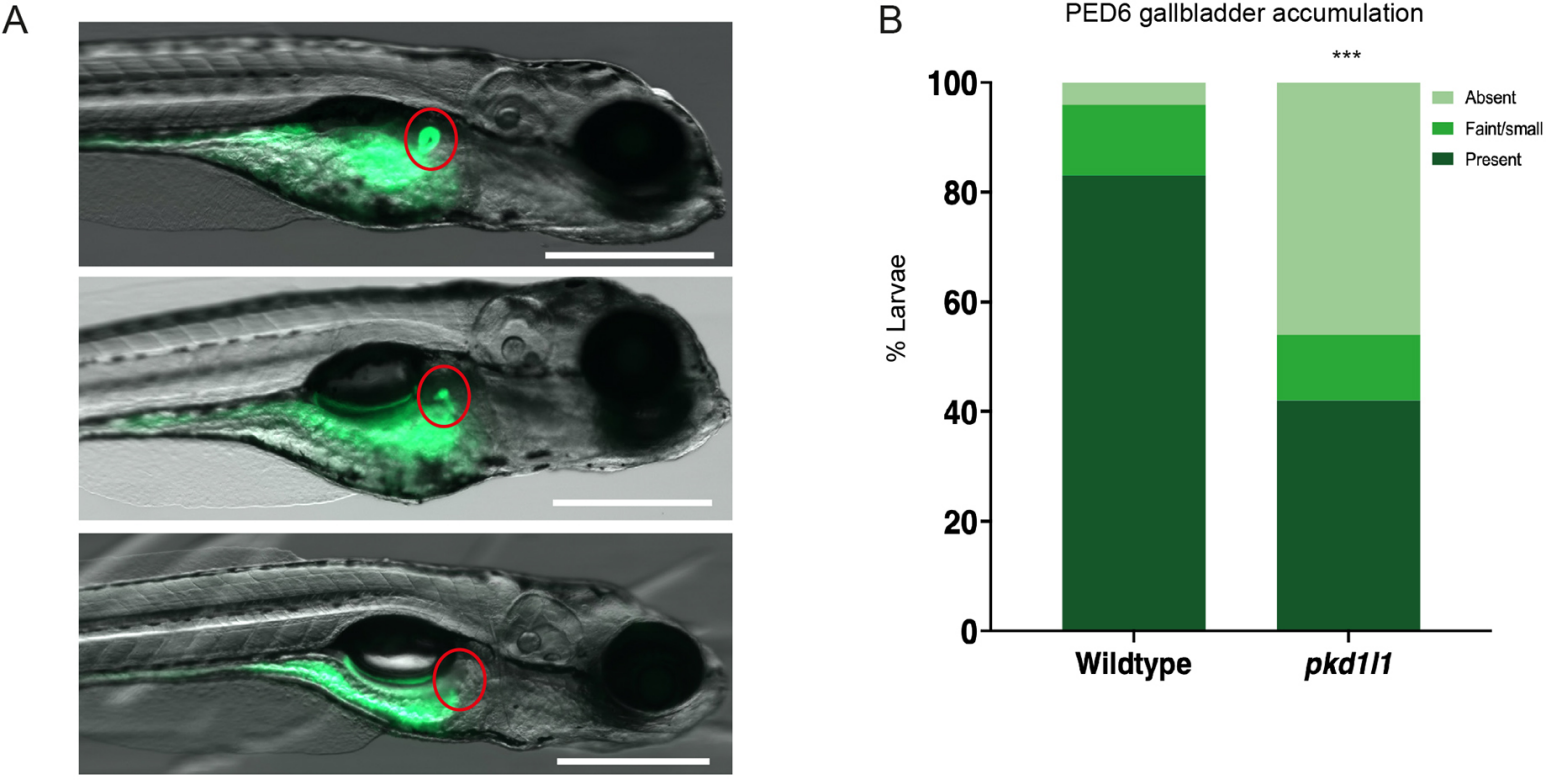
***pkd1l1* knockout alters hepatobiliary function.** (A) Representative images of PED6 uptake in larvae showing typical normal (top), faint (middle) and absent (bottom) fluorescence (red circles) in gallbladders. Scale bars: 500 µm. (B) Quantification of PED6 uptake showed an increase number of absent gallbladders in *pkd1l1* mutant (*n*=145) compared to wild-type (*n*=84) larvae (***P<0.001; χ^2^ test), reflecting defects in biliary function.

In a functioning and structurally intact zebrafish hepatobiliary system, the fluorescently labeled phospholipid PED6 can be ingested, absorbed by the intestine, transported to the liver and excreted into bile. PED6 is then transported through the intrahepatic biliary network, before accumulating in the gallbladder. Decreased PED6 uptake in the zebrafish gallbladder is a strong indicator of impaired hepatobiliary function (Farber et al., 2001; Matthews et al., 2008; Cui et al., 2011; Tang et al., 2016). PED6 accumulation in the gallbladder was significantly reduced in *pkd1l1^hsc117^* mutant larvae (46%), compared to in wild-type larvae (4%) (Fig. 3A,B). Taken together, these data suggest that Pkd1l1 function is required for normal hepatobiliary function.

### Pkd1l1 is involved in intrahepatic bile duct development

**Fig. 4.**
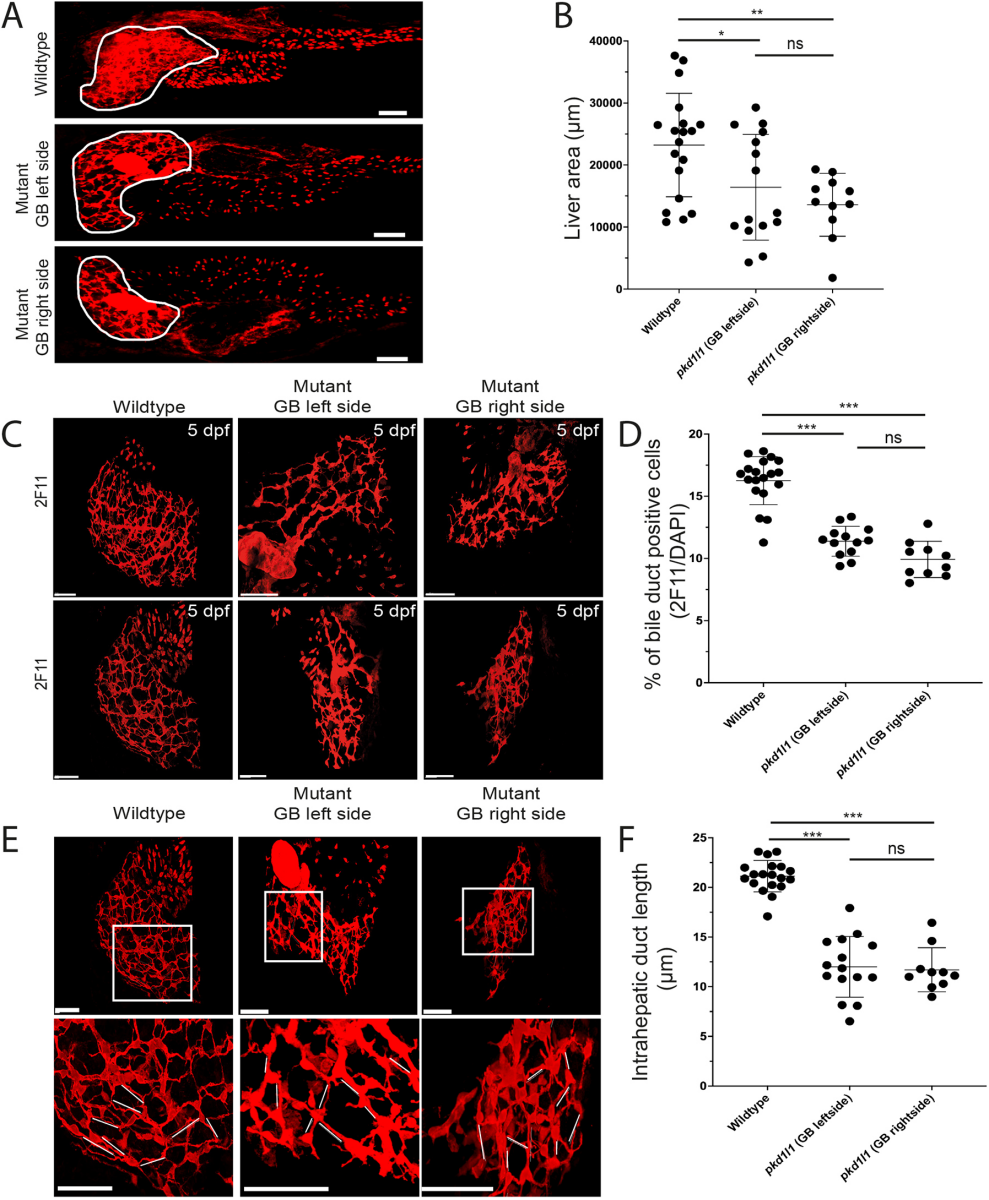
**Pkd1l1 function is necessary for normal bile duct development.** (A) Confocal images showing bile ducts (assessed by 2F11 antibody staining) in liver (white outline) in wild type and mutants. GB, gallbladder. (B) Quantification showed reduction of the area of the liver in mutants compared to that in the wild type. (C,D) Whole-mount immunostaining of zebrafish livers (C) showed a decrease number of biliary epithelial cells (BECs) and abnormal intrahepatic biliary network in *pkd1l1* mutant larvae compared to those in wild-type larvae (D). The numbers of BECs were measured by counting 2F11 and DAPI double-positive cells and the percentages were calculated by dividing by the total number of DAPI-positive cells from each fish. (E,F) Confocal images (E) showed a reduced length of intrahepatic ducts (white lines) in mutants compared to that in the wild type. The lengths of intrahepatic ducts were calculated by measuring the duct distance between two cell bodies and the average of ten ducts was calculated per image (fish) (F). Graphs show the mean±s.d. Scale bars: 50 µm. ns, not significant; *P<0.05; **P<0.01; ***P<0.001 (one-way ANOVA with Tukey post hoc test).

We assessed whether absence of Pkd1l1 leads to abnormal development of a bile duct network. As shown above, *pkd1l1^hsc117^* mutants did not exhibit gross morphological differences compared to the wild type; however, their liver area, assessed by the 2F11 antibody, appeared smaller compared to that of the wild type (Fig. 4A,B). In wild-type larvae, whole-mount staining revealed a high number of biliary epithelial cells (BECs) as well as bile ducts that interconnect BECs to make a well-defined and dense network of ducts. In contrast to wild-type larval livers, *pkd1l1^hsc117^* mutant larval livers had fewer BECs and a reduced number of interconnecting ducts. We also noted that the bile ductal network was poorly defined with a reduced density in *pkd1l1^hsc117^* mutant livers compared to that in wild-type livers (Fig. 4C,D). Furthermore, the length of the interconnecting ducts, as measured by the distance between nuclei of two BECs, was significantly reduced in mutants compared to in wild types (Fig. 4E,F). These data suggest that mutant Pkd1l1 expression affects the development of the biliary tree in the zebrafish liver.

### Pkd1l1 deficiency alters liver enzyme activity

**Fig. 5.**
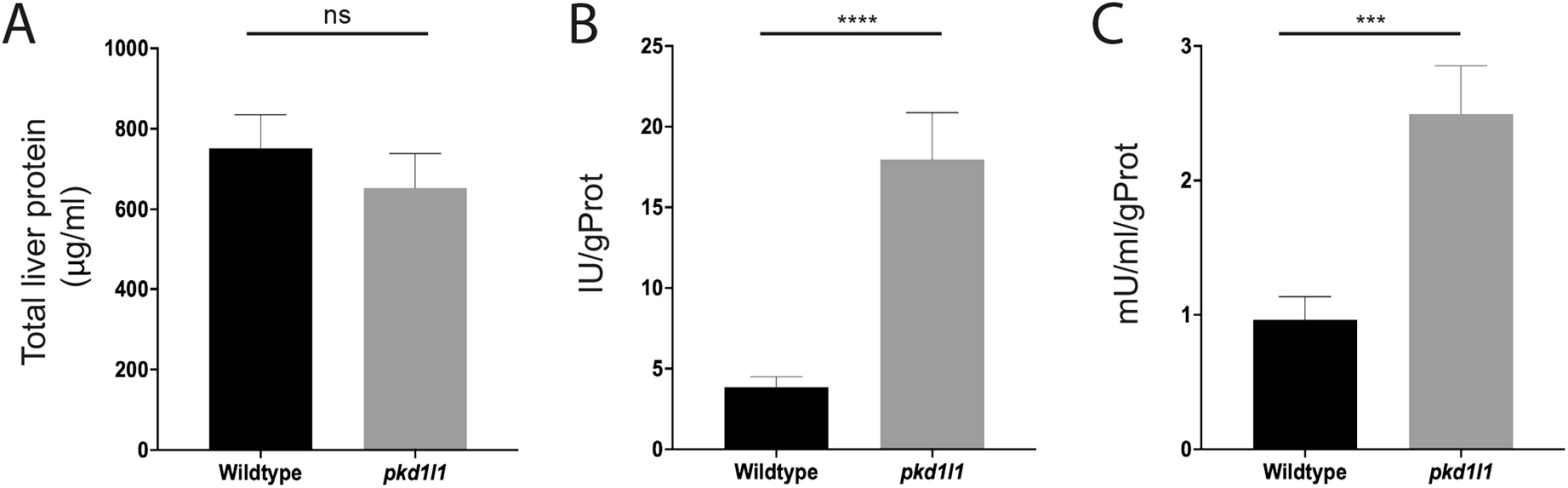
***pkd1l1* mutant zebrafish have abnormal liver biochemistry.** Whole livers were isolated from 6-week-old zebrafish and homogenized for protein assays. (A) Total liver protein concentration showed no significant differences between wild-type and *pkd1l1* mutant livers (*n*=16). (B,C) ALT (B) and GGT (C) enzyme assays showed a significant increase in enzyme activity in *pkd1l1* mutant livers compared to that in wild-type livers (*n*=15). Graphs show the mean±s.e.m. ns, not significant; ***P<0.001; ****P<0.0001 (unpaired two-tailed Student's *t*-test).

We assayed alanine aminotransferase (ALT) and γ-glutamyl transferase (GGT) activity in whole livers from *pkd1l1^hsc117^* mutant zebrafish at 6 weeks post fertilization. Quantification of total liver protein showed no significant difference in protein concentration in wild-type and *pkd1l1^hsc117^* mutant livers (Fig. 5A). ALT activity was significantly increased in *pkd1l1^hsc117^* mutant livers (17.98 IU/g protein) compared to that in wild-type livers (3.85 IU/g protein) (*P*<0.0001) (Fig. 5B). GGT activity was also increased significantly in *pkd1l1^hsc117^* mutant livers (2.49 mU/ml/g protein) compared to that in wild-type livers (0.96 mU/ml/g prot) (*P*<0.01) (Fig. 5C). Taken together, these analyses suggest that the absence of normal PKD1L1 functioning is associated with increased liver enzyme activity.

## DISCUSSION

In this study, we demonstrate a previously unreported role for Pkd1l1 in hepatobiliary development. Knockout of *pkd1l1* in zebrafish by CRISPR/Cas9 editing led to developmental biliary anomalies and impaired hepatobiliary function. In addition, *pkd1l1^hsc117^* mutant zebrafish demonstrated laterality defects of the gallbladder, consistent with the known role of Pkd1l1 in L-R patterning.

PKD1L1 belongs to the PKD1 family, the interaction of which with PKD2 during embryonic development drives normal L-R patterning (Field et al., 2011; Kamura et al., 2011). Although the ciliary morphology and motility of nodal cilia were normal in a study of *Pkd1l1* and *Pkd2* mutant mice, the authors suggested a downstream role of these two genes in nodal flow and hypothesized that these interactions sense nodal flow and are involved in left-side activation of the nodal signaling cascade (Field et al., 2011). In medaka, mutations in the homolog of *PKD1L1* also result in laterality defects, similar to our findings (Kamura et al., 2011). Deletion and/or mutation of *Pkd1l1* in mice resulted in reduced viability during embryonic development or the early postnatal period (Vogel et al., 2010; Field et al., 2011). Vogel et al. (2010) hypothesized that the reduced viability in mice is associated with undiagnosed congenital heart disease. We and others have also showed that heart looping and heart development is affected by Pkd1l1; however, none of these studies have examined congenital heart disease in these animal models (Field et al., 2011; Kamura et al., 2011).

Complementary to a recently published mouse model outlining the fibroinflammatory response to *Pkd1l1* deletion in developing livers (Hellen et al., 2023), this study is the first to show a functional role for Pkd1l1 in the hepatobiliary system of a model organism. These two studies build upon the identification of *PKD1L1* as a candidate gene for BASM using whole-exome sequencing in a large cohort of patients with BASM (Berauer et al., 2019). This study identified nine deleterious variants in *PKD1L1*. One of the variants identified in this study, a splice site mutation (c.6473+2_6473+3delTG), was previously reported by Vetrini et al. (2016) in two siblings with heterotaxy and severe congenital heart disease. However, it is not known whether these two siblings had splenic or biliary tract abnormalities. Berauer et al. (2019) also showed that PKD1L1 is strongly expressed in the bile duct epithelium in liver tissue collected from an unaffected infant and two patients affected by non-cholestatic liver disease. Of note, the expression of PKD1L1 was weak or absent in liver tissue from a patient with BASM. The specific molecular roles for *PKD1L1* in mammalian cholangiocyte development and BA are currently unknown.

Our data indicate that Pkd1l1 deficiency results in impaired development of a biliary duct network and reduced bile accumulation in the gallbladder of mutant zebrafish larvae. These results strongly support the need for further studies to dissect the mechanism as to how loss of PKD1L1 signaling may result in hepatobiliary disease in humans. Previous studies in cultured cells have shown that Pkd1l1 interacts with Pkd2l1 in primary cilia, to form a ciliary calcium channel and regulate hedgehog signaling (DeCaen et al., 2013; Delling et al., 2013). Cholangiocytes possess cilia that extend from the apical plasma membrane into the bile duct lumen and act as cellular antennae to detect and modify bile flow, osmolarity and composition (Masyuk et al., 2008). Cilia play an important role in the regulation and transmission of downstream intracellular signaling pathways (Braun and Hildebrandt, 2017). We hypothesize that Pkd1l1 maintains intraciliary calcium signaling in cholangiocytes and alterations of this signaling contribute to structural and functional defects in cholangiocytes. Direct testing of this and other signaling hypotheses for Pkd1l1 await development of appropriate reagents and models.

To further support the hypothesis that Pkd1l1 signaling is required for hepatobiliary development, it would be ideal to document expression of this protein at different stages of development in zebrafish. However, like many ciliary genes, *pkd1l1* is expressed at very low levels. A review of publicly available single-cell gene expression data from the zebrafish database Daniocell did not reveal *pkd1l1* RNA expression in the endoderm or liver at any stage of zebrafish development (3-120 hpf), which is perhaps not surprising given the known sequencing-depth limitations of single-cell RNA sequencing. We therefore believe that it is not feasible to identify *pkd1l1* expression in the zebrafish liver with current technologies. This inability to identify genes involved in hepatobiliary development is not a feature unique to *pkd1l1*. Knockdown of other BA ciliary candidate genes, such as *kif3b* and *ttc17*, resulted in abnormal biliary tract development in zebrafish (Lam et al., 2021); however, these are also not yet reported to be expressed at any time point in the liver during zebrafish development, according to the Daniocell database. Taken together, it is apparent that there are a number of genes with low expression such as *pkd1l1* and *gpc1b* that are crucial for biliary tract development in zebrafish, yet their expression levels are below the limits of detection using current technologies. In addition, published proteomic analyses of cilia demonstrate that ciliary signaling molecules such as the PKD1L1/PKD1L2 channels are expressed in extremely low quantities (∼150 copies/cell), which would made detection of Pkd1l1 virtually impossible with standard immunohistochemical and molecular approaches (DeCaen et al., 2013; Mick et al., 2015).

Increased levels of liver enzymes are a typical marker of liver disease in humans and have also been associated with disease conditions in the zebrafish model (Rangasamy et al., 2018; Bao et al., 2020; Batista-Silva et al., 2020; Derikvandy et al., 2020). In the current study, high levels of the liver enzymes ALT and GGT were detected in whole-liver homogenates of *pkd1l1* mutant zebrafish compared to those in wild type. In clinical practice, these enzymes are assayed in the serum, and their levels reflect liver cell damage and leakage of these enzymes into the bloodstream. Although serum levels were not assayed in our study as in clinical practice, it is possible that these significantly elevated levels in liver homogenates indicate some intrinsic hepatocellular and biliary damage within the mutant zebrafish livers, potentially as a result of retained bile flow, which we also demonstrated. This, of course, is comparable to the situation in human BA.

In conclusion, our study demonstrates that mutation of *pkd1l1*, previously identified by whole-exome sequencing as a candidate susceptibility gene for BASM, resulted in defective biliary structure and impaired hepatobiliary function in zebrafish larvae. This is consistent with recent mouse data and, taken together, strongly support the role of *pkd1l1* as a disease-susceptibility gene for BASM. These data also shed light on a potential mechanism wherein deranged ciliary signaling can result in hepatobiliary damage. This study lays the foundation for additional validation in mice and human-derived tissue, likely using induced pluripotent stem cell technology.

## MATERIALS AND METHODS

### Fish maintenance and husbandry

Established zebrafish husbandry protocols were adhered to and all protocols were performed in accordance with the Canadian Council on Animal Care guidelines. Wild-type zebrafish from AB and TU strains were used. Embryos from natural matings were maintained at 28°C. When required, zebrafish were euthanized with tricaine (500 mg/l; MS-222/MESAB, Sigma-Aldrich, E10521), followed by submersion of anesthetized fish in ice water for several minutes.

### CRISPR/Cas9 targeting of *pkd1l1*

The *pkd1l1^hsc117^* allele was generated by the SickKids Zebrafish Core Facility using CRISPR/Cas9 mutagenesis (Armstrong et al., 2016). Exon 3 of *pkd1l1* was targeted with sgRNAs (5′-ACAAGCTCTGATCTGCCAGT-3′) designed using CHOPCHOP (Montague et al., 2014; Labun et al., 2016). Briefly, 50 pg sgRNA was injected into the single-cell zebrafish embryos, along with 150 pg *cas9* RNA. Mosaic F0 embryos were reared to adulthood and outcrossed to wild-type (AB) fish, and the resulting F1 embryos were screened for germline *pkd1l1* mutations using high-resolution melt analysis (Parant et al., 2009). Sanger DNA sequencing, which was performed by The Centre for Applied Genomics at SickKids, confirmed isolation of a four-base-pair deletion (Δ4 bp) allele designated *pkd1l1^hsc117^*. Sequence analysis was performed using Geneious (Kearse et al., 2012).

### *In situ* hybridization

Motile cilia dysfunction often presents with abnormal L-R axial patterning (Essner et al., 2005). To test laterality, left-sided *lefty1/2* expression and directionality of cardiac looping, using the marker cardiac myosin light chain 2 (*cmlc2*), was assayed (Yelon et al., 1999; Chen and Shen, 2004; Cheng et al., 2004). Antisense RNA probes were prepared using a digoxigenin RNA labeling kit (Roche) from linearized DNA. Whole-mount RNA *in situ* hybridizations were performed according to standard protocols (Thisse et al., 1993). Briefly, samples were dechorionated and fixed in 4% paraformaldehyde at 4°C overnight. Probes were added at 1 ng/µl in hybridization buffer [50% formamide, 20× SSC, 100 µg/ml heparine (Sigma-Aldrich, disodium salt), 1 mg/ml Torula RNA (Sigma-Aldrich), 0.1% Tween 20, 1 M citric acid to adjust pH to 6.0-6.5 and water] to samples and incubated in a 68°C water bath overnight. Embryos were washed at 68°C for 10 min in 75% [50% formamide/5× saline-sodium citrate (SSC) buffer]/25% 2× SSC, 10 min in 50% (50% formamide/5× SSC)/50% 2× SSC, 10 min in 25% (50% formamide/5× SSC)/75% 2× SSC, 10 min in 2× SSC, and two times for 30 min in 0.2× SSC/0.01% Tween-20. Further washes were performed at room temperature for 10 min in 75% 0.2× SSC/25% PBS containing 0.25% Triton X-100 (PBST), 10 min in 50% 0.2× SSC/50% PBST, 10 min in 25% 0.2× SSC/75% PBST, 5 min in PBST and 5 min in malate buffer (100 mM maleic acid, 150 nM NaCl, 0.1% Tween 20, water to 1 l and NaOH to adjust pH to 7.5). Samples were blocked in malate buffer with 20% 5× blocking stock reagent and 2% heat-inactivated sheep serum at room temperature for 4 h. Embryos were incubated overnight at 4°C with anti-digoxigenin-AP Fab fragments (Sigma-Aldrich) in malate buffer with 20% 5× blocking stock reagent at a 1:1000 dilution. Finally, embryos were washed eight times for 15 min each in PBST at room temperature. Detection was performed using alkaline phosphate NBT-BCIP solution (Roche, 11681451001). Once the color developed, the reaction was stopped with one PBST wash. Samples were stained in the same tube to control for possible variations in signal strength and were genotyped after imaging.

### RNA microinjections in embryos and live imaging of KVs

Microinjection of mRNA in embryos and live imaging of KVs were performed according to the protocol detailed by Borovina et al. (2010). Briefly, mRNA for microinjections was synthesized *in vitro* using the mMessage mMachine transcription kit (Invitrogen) from linearized plasmids encoding memRFP and green fluorescent protein-tagged Arl13b (Arl13b-GFP). Injections were performed on wild-type and *pkd1l1^−/−^* embryos at the one-cell stage. Embryos were injected with 10 pg memRFP- and 7 pg Arl13b-GFP-encoding plasmids. The embryos were placed in a 28°C incubator until the shield stage was reached, after which they were placed at 23°C until the 10-somite stage. At the 10-somite stage, embryos were dechorionated and mounted in low-melt 0.8% agarose for live imaging. KV imaging was performed using a Zeiss 710 laser scanning confocal microscope at 63× magnification. Images were line-averaged over four scans to produce the blurred, fanlike appearance of motile cilia. *Z*-stack projections were assembled using ImageJ software.

### PED6 treatment

To test the hypothesis that Pkd1l1 is involved in hepatobiliary function, we measured gallbladder accumulation of fluorescently labeled PED6. Wild-type and *pkd1l1^hsc117^* mutant larvae at 5 dpf were soaked for 3 h in 0.3 µg/ml PED6 (Thermo Fisher Scientific), a fluorescently labeled or quenched phospholipid, to allow visualization of the gallbladder, as previously described (Farber et al., 2001). After 3 h treatment, larvae were anesthetized with tricaine and gallbladder images were scored as normal, faint or absent, based on fluorescence intensity in images acquired using an Axio Zoom.V16 microscope (Zeiss). Positioning of the gallbladder was scored as left or right.

### Immunostaining of zebrafish larvae

To study the architecture of the biliary tree, 5 dpf zebrafish larvae were stained with the 2F11 antibody (Abcam, ab71286). Larvae were fixed with 4% paraformaldehyde overnight and then in 100% ice-cold methanol for long-term storage. Prior to antibody treatment, larvae were rehydrated through decreasing concentrations of methanol in PBS containing 0.2% Triton X-100 (PBSX). The skin was then peeled off the larvae and they were deyolked and treated with collagenase 1 (1 mg/ml, Stem Cell Technologies, 07415) for 15 min at room temperature. Blocking was performed for 1 h with 10% bovine serum albumin with 0.2% Triton X-100. The 2F11 antibody (1:100) was diluted with blocking reagents and incubated overnight at 4°C on a shaker. The next day, larvae were washed with PBSX and incubated with secondary antibody (goat anti-mouse Cy3, 1:500, Jackson ImmunoResearch, 115-166-071) overnight at 4°C on a shaker. Whole larvae were mounted on glass slides with anti-fade reagent (Dako, S3023). Images of immunostained zebrafish larvae were acquired using a Leica SP8 light-sheet confocal microscope. Confocal stack images were processed and analyzed using Imaris software (Bitplane). The length of intrahepatic ducts, liver area and number of BECs were measured using ImageJ software.

### Liver enzyme analysis

Liver tissue was collected from 6-week-old wild-type and *pkd1l1^hsc117^* mutant zebrafish in PBS (0.01 M, pH ∼7-7.4) at a ratio of 1 g tissue/9 ml PBS (w/v). To extract total protein, liver tissue was homogenized on ice by sonication and centrifuged at 10,000 ***g*** for 10 min. Protein concentration in the lysates was determined using a Pierce BCA protein assay Kit (Thermo Fisher Scientific, 23227). Liver enzymes were measured using an ALT assay kit (Elabscience, E-BC-K235-M) and a GGT activity colorimetric assay kit (BioVision, K784-100) according to the manufacturers' protocols.

### Statistical analysis

For the *in situ* hybridization, PED6 treatments and presence of gallbladder experiments, statistical analyses were performed using χ^2^ testing. For measuring lengths of intrahepatic ducts, areas of livers and numbers of BECs, one-way ANOVA with Tukey post hoc test was performed. For the ALT and GGT assays, unpaired two-tailed Student's *t*-tests were performed. For all analyses, *P*<0.05 was considered statistically significant.
